# Radiology and clinical prognosis of posterior malleolar fractures with different Step-off levels

**DOI:** 10.1186/s12891-025-09050-8

**Published:** 2025-08-16

**Authors:** Yongqi Li, Rui Luo, Bing Li, Hongzi Wu, Zongbiao Bai, Yong Jin, Yunfeng Yang

**Affiliations:** 1 Department of Orthopedics, Karamay Hospital of People’s Hospital of Xinjiang Uygur Autonomous Region(Central Hospital of Karamay), Karamay, 834000 China; 2 Department of Orthopaedics, Ruijin Hospital, Shanghai Jiao Tong University School of Medicine, Shanghai, 200025 China; 3 Department of Neurology, Karamay Hospital of People’s Hospital of Xinjiang Uygur Autonomous Region(Central Hospital of Karamay), Karamay, 834000 China

**Keywords:** Posterior malleolar fracture, Step-offs, Post-traumatic osteoarthritis, Functional prognosis

## Abstract

**Objective:**

Ankle fracture involving the posterior malleolus is characterized by poor prognosis, and its risk factors remain controversial. This study aims to evaluate the influence of posterior malleolar fractures with different step-off levels on posttraumatic osteoarthritis and functional prognosis and identify the related risk factors that affect the clinical prognosis of posterior malleolar fractures.

**Methods:**

The information of 134 patients with ankle fractures involving the posterior malleolus from January 2016 to December 2021 was retrospectively collected. The patients’ posterior malleolar fracture reduction quality, fracture healing status, and severity of ankle posttraumatic osteoarthritis (Kellgren–Lawrence scale) were evaluated by radiology, and the ankle function was assessed using Olerud-Molander score, visual analogue scale (VAS) score, and range of ankle motion.

**Results:**

All 134 patients achieved fracture healing, with an average follow-up period of 42 (13,78) months. Thirty-nine patients (29%) showed positive ankle posttraumatic osteoarthritis. The average Olerud-Molander score was 89.9 (70,100), the average VAS score was 1.0 (0,5), and the ankle dorsiflexion restriction was 4.0° (0°,9°) on the average. Compared with the proportion of patients with postoperative step-off of < 1 mm of posterior malleolar fracture, the proportion of patients with positive posttraumatic osteoarthritis at postoperative step-offs of 1–2 and > 2 mm was higher (*P* < 0.05), and the functions of the affected limbs were evidently degraded Olerud-Molander score, VAS score, and ankle dorsiflexion restriction; *P* < 0.05). However, the differences in the proportion of patients with positive posttraumatic osteoarthritis and functional prognosis between postoperative step-offs of 1–2 mm and > 2 mm were not statistically significant (*P* > 0.05). Posterior malleolar step-off was an independent, statistically significant risk factor for posttraumatic osteoarthritis (Wald = 14.23, *P* < 0.01), and both posterior malleolar step-off and posttraumatic osteoarthritis were independent risk factors that led to the poor functional prognosis (*P* < 0.05).

**Conclusion:**

Clinically, importance should be given to the anatomical reduction of posterior malleolar fractures and the incidence of posttraumatic osteoarthritis to improve the long-term functional effects on affected limbs.

**Fund program:**

Natural Science Foundation of Xinjiang Uygur Autonomous Region（2024D01C11; Xinjiang Tianshan Talent Training Program (2023TSYCJC0053); Shanghai Science and Technology Commission Project (22S31900300, 21ZR1458500).

The posterior malleolus is a part of the distal tibiofibular complex, which plays an important role in the contact area of the tibiotalar joint, the bearing capacity of the human body, and the stability of the ankle [1, 2]. The proportion of ankle fractures in posterior malleolar fractures is high (7–44%), and the prognosis of partial ankle fracture involving posterior malleolus is relatively poor [3–5].

Currently, there remains controversy among some researchers regarding the surgical fixation indications and prognostic risk factors for posterior malleolus fractures [6–8]. Drijfhout van H et al. [9] reported that the incidence of radiographic posttraumatic osteoarthritis in patients with moderate and large posterior malleolar fractures and postoperative step-offs ≥ 1 mm is high. Abarquero-Diezhandino A. et al. [10] stated that large posterior malleolar fragments are associated with severe early posttraumatic osteoarthritis (*P* < 0.03) and poor Olerud-Molander score (*P* < 0.01). Neumann AP [11] posited that the size of a posterior malleolar fragment has no prognostic value. The risk factors that affect the prognosis of posterior malleolar fractures reported in previous related studies differ, and the conclusions are inconsistent or even contradictory [12, 13]. The effects of the size of the posterior malleolar fragment and the step-offs of posterior malleolar fractures are controversial. However, the difference in the prognosis of posterior malleolar fractures with different step-off levels has been rarely reported.

In this study, the clinical and imaging data of 134 patients with ankle fractures involving the posterior malleolus were retrospectively collected and analyzed to (1) evaluate the influence of posterior malleolar fractures with different step-off levels on the radiological results (posttraumatic osteoarthritis) and functional prognosis Olerud-Molander score, VAS score, and ankle dorsiflexion restriction) and (2) explore and identify the related risk factors that affect the clinical prognosis of posterior malleolar fractures reported in previous studies to guide clinical diagnosis and treatment.

## Materials and methods

### Inclusion and exclusion criteria

The inclusion criteria were as follows: ankle fractures involving the posterior malleolus; patients not younger than 18 and not older than 70; the posterior malleolar fracture had been surgically fixed (plate or screw); complete follow-up data(the follow-up period lasted for at least 12 months).

The exclusion criteria were as follows: old ankle fracture, pathological ankle fracture, and open ankle fracture, ankle and foot deformities before trauma.

**Fig. 1:**
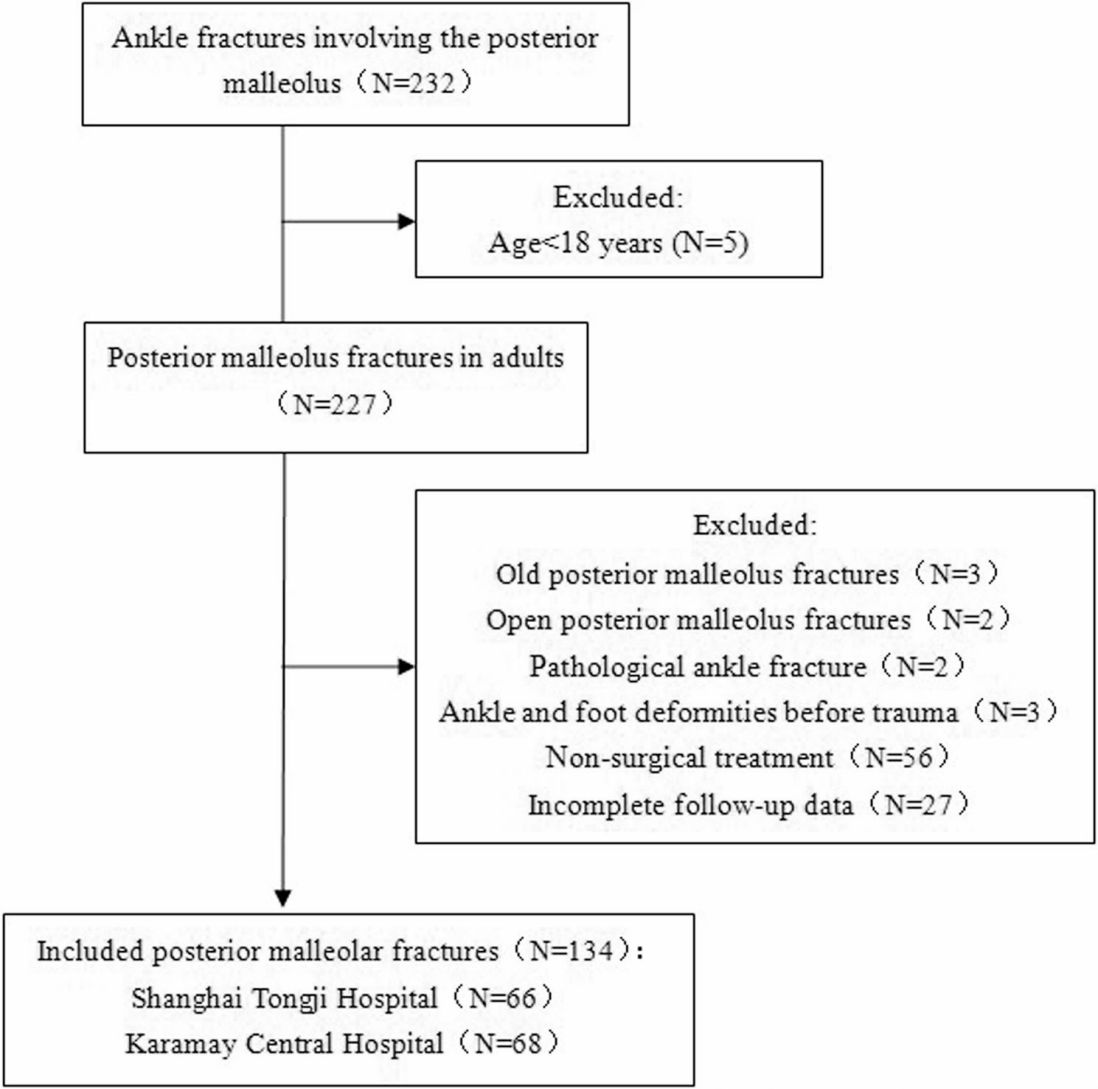
Flowchart of patient selection

A total of 134 cases with ankle fractures involving the posterior malleolus from January 2016 to December 2021 were collected (Fig. 1). This study was approved by the Medical Ethics Committee of Tongji Hospital of Tongji University (KW-2021-015).

### Operative technique

The posterolateral approach was adopted for Haraguchi-1 posterior malleolar fracture [14], which was exposed and reduced through the space between flexor hallucis longus and peroneal longus muscles and fixed with posterior–anterior lag screws or buttress plates. During this period, attention was paid to protecting the sural nerve and the small saphenous vein. The posterolateral approach was applied to Haraguchi-2 A type, and the posterolateral approach combined with the posteromedial approach was used for Haraguchi-2B type. The posterolateral approach was the same as before. For the posteromedial approach, the posteromedial fragment was exposed between the flexor digitorum longus muscle and the posterior tibial neurovascular bundle. After reduction, the posterior malleolar fracture was fixed using posterior–anterior lag screws or buttress plates, and the posterior tibial neurovascular bundle was protected. All operations were performed by the clinically experienced associate chief physician or the chief physician of the Foot and Ankle Surgery Department.

The isometric contraction exercise of quadriceps femoris and calf muscles began on the second day after operation. The non-weight training exercise lasted for at least six weeks until a sign of radiographic fracture healing appeared. The patients were instructed to start partial weight training six weeks after the operation and strive to complete the weight training by the 12th week.

### Radiographic measurement

**Fig. 2:**
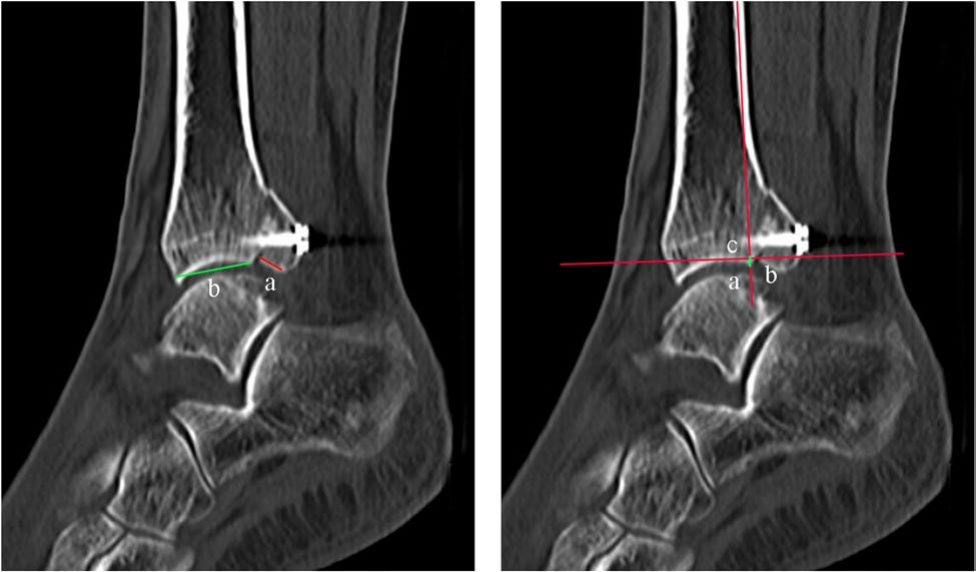
CT radiographic measurement of patirents. **A** Percentage of posterior malleolar fractures on the articular surface: a/(a+ b) × 100% (**a**. length of the articular surface of the posterior malleolar fragment; **b**. length of the articular surface of distal tibia). **B** Posterior malleolar fracture step-off: ac distance (**a**. fracture point of the articular surface of distal tibia; **b**. fracture point of the articular surface of the posterior malleolar fragment; **c**. intersection between the long axis passing point a and being parallel to tibia and the long axis passing point b and being perpendicular to tibia)

After the operation, X-ray and Computed Tomography (CT) examinations of the ankle were completed. The percentage of the posterior malleolar fracture on the articular surface and the step-off level of the posterior malleolar fracture were measured by referring to the methods of Lee S. H. et al. [15] and Marques Ribeiro H. et al. [16], respectively. The maximum value of the weight bearing CT sagittal plane was adopted as the measurement standard (Fig. 2). All data were measured twice respectively by the same clinically experienced associate chief physician of the Foot and Ankle Surgery Department, and the average value was considered as the final value.

### Radiographic evaluation

As suggested by Ketz et al. [17], the reduction quality of a posterior malleolar fracture was evaluated by postoperative CT scanning and classified as follows: anatomical reduction = step-off < 1 mm, favorable reduction = step-off of 1–2 mm, and poor reduction = step-off > 2 mm.

Follow-up patient visits were performed to determine the fracture healing status of the patients. Radiographic healing means that the bridging callus is formed in at least three layers of cortical bone, or the fracture line appears [7].

**Fig. 3:**
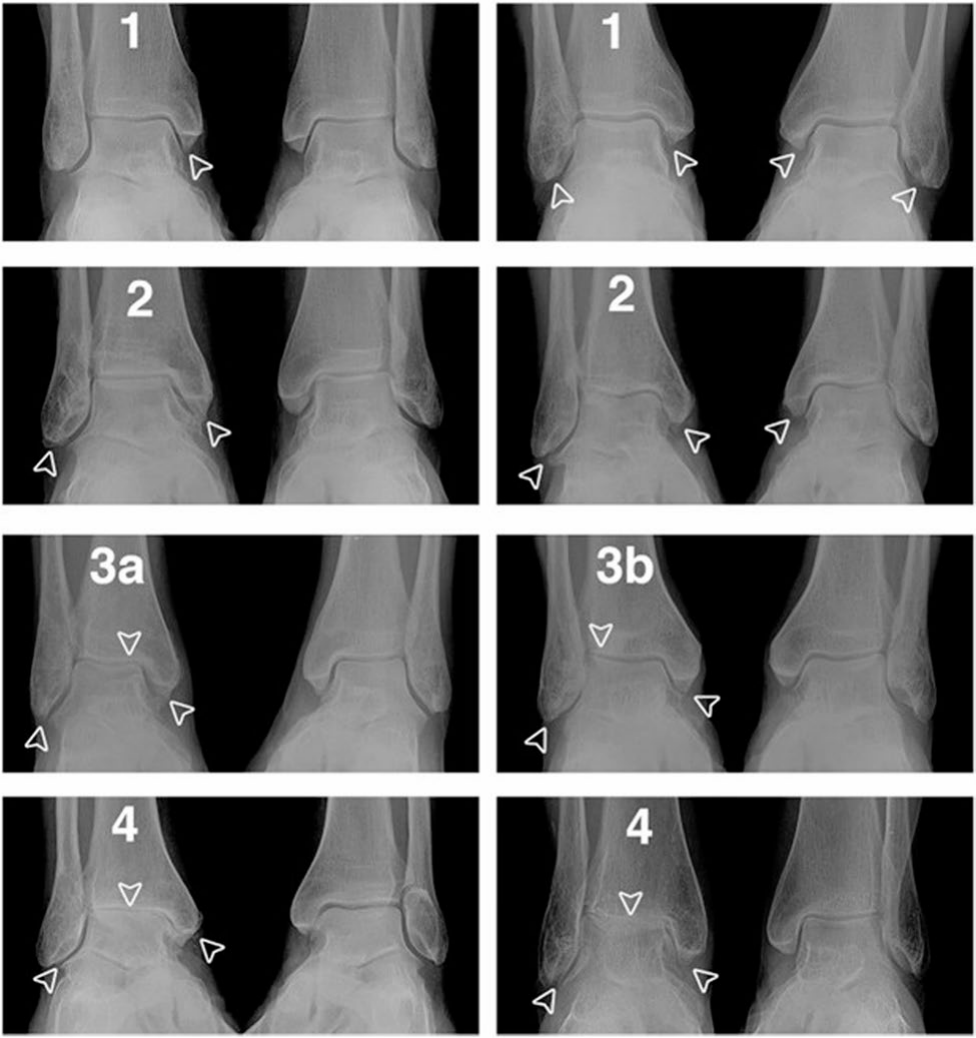
Image examples of K&L scale for ankle OA. Two examples are displayed for each grade with operated side of relevant grade on the left and contralateral side without fracture on the right. Grade 1: example 1-operated side displays osteophytes of doubtful significance on the medial malleolus. Example 2-operated and non-operated sides display osteophytes of doubtful significance on the medial and lateral malleoli. Grade 2: example 1-operate side displays definite osteophytes on the medial and lateral malleoli. Example 2-operated side displays definite osteophytes on medial and lateral malleoli; contralateral side displays osteophytes of doubtful significance on the medial malleoli. Grade 3a: operated side displays definite osteophytes on the medial and lateral malleoli, moderate (<half) joint space narrowing and tibiotalar tilt 2° (1.6°). Grade 3b: operated side displays definite osteophytes on the medial and lateral malleoli, moderate (<50%) medial joint space narrowing and tibiotalar tilt 2° (3.3°). Grade 4: example 1-operated side displays definitive osteophytes on the medial and lateral malleoli as well as prominent (>50%) joint space width narrowing and tibiotalar sclerosis. Example 2-operated side displays definitive osteophytes on the medial and lateral malleoli as well as complete joint space width narrowing and tibiotalar sclerosis.(From Holzer N, Salvo D, Marijnissen A, et al. [18])

In the last follow-up visit, X-ray films of the front lateral position of the ankle and ankle acupoints were obtained, and the severity of the osteoarthritis/posttraumatic osteoarthritis of the ankle was evaluated with the Kellgren–Lawrence scale (Fig. 3) [18], where Grade 0 means normal; Grade 1 denotes suspected osteophyte formation and suspected joint space stenosis, Grade 2 refers to mild osteophyte formation and suspected joint space stenosis; Grade 3 means moderate osteophyte formation, narrow joint space, and suspected bone deformity; and Grade 4 covers severe osteophyte formation, obvious joint space stenosis, severe bone sclerosis, and deformity. Grade 0 was considered negative posttraumatic osteoarthritis, and Grades 1–4 indicated positive posttraumatic osteoarthritis.

### Clinical evaluation

The ankle joint function was evaluated using the Olerud-Molander score, and the full score was 100.

Ankle pain was evaluated using VAS with scores ranging from 0 to 10, where 0 represents no pain and 10 indicates unbearable, sharp pain.

The range of motion of bilateral ankles was measured [9], and the difference in the dorsiflexion of bilateral ankles was defined as ankle dorsiflexion restriction.

#### Statistical analysis

With SPSS 25.0 statistical software, the measurement data were subjected to normality and homogeneity tests of variance on the basis of Kolmogorov–Smirnov and Levene methods. The data that conformed to a normal distribution were expressed by x ± s and subjected to ANOVA. The data that did not conform to a normal distribution were expressed by M (Q1, Q3), and the Kruskal–Wallis H test was performed. The radiological results (posttraumatic osteoarthritis) of posterior malleolar fractures with different step-off levels were analyzed using the chi-squared test. The post hoc test was performed using partitions of the chi-square. Multiple comparison corrections were applied using Bonferroni method. Logistic and linear regression analyses were conducted on the radiographic results (posttraumatic osteoarthritis) and functional prognosis (Olerud-Molander score), respectively (the multivariate regression analysis was conducted for those with *P* < 0.20 after the univariate analysis). *P* < 0.05 indicated that the difference was statistically significant.

## Results

### General information

In this study, 68 cases were from Karamay Central Hospital (50.7%), and 66 cases were from Shanghai Tongji Hospital (49.3%). Among them, 56 (41.8%) and 78 (58.2%) cases were males and females, respectively, with an average follow-up period of 42 (13,78) months. In addition, anatomic reduction was achieved in 88 cases(65.7%) of posterior malleolus fractures, and 103 cases (76.9%) presented an area ratio of posterior malleolar fragments > 25%. The baseline characteristics of the patients are listed in Table 1.

**Table 1:** Basic characteristics of patients

Basic characteristics	
Number of patients, N	134
Follow-up in months, mean (range)	42.0(13,78)
Age in years, mean (range)	48.7(18,70)
Haraguchi classification, N(%)	
Haraguchi-1	87(64.9)
Haraguchi-2 A	13(9.7)
Haraguchi-2B	34(25.4)
Posterior malleolar step-off, N(%)	
<1 mm	88(65.7)
1–2 mm	30(22.4)
>2 mm	16(11.9)
Area ratio of the posterior malleolar fragment, N(%)	
<25%	31(23.1)
>25%	103(76.9)
Fracture type *, N(%)	
P	5(3.7)
PL	19(14.2)
PLM-SER	96(71.6)
PLM-PER	14(10.4)
Kellgrene–Lawrence scale of posttraumatic osteoarthritis, N(%)	
0	95(70.9)
1	18(13.4)
2	14(10.4)
3	7(5.2)
4	0(0)
Olerud-Molander, mean (range)	89.9(70,100)
VAS, mean (range)	1.0(0,5)
Ankle dorsiflexion restriction(°), mean (range)	4.0(0,9)

^*^P, Pure posterior malleolus fracture; PL, Bimalleolar fracture (posterior malleolus + lateral malleolus/fibular fracture); PLM-SER, Trimalleolar fracture-supination external rotation (posterior malleolus + lateral malleolus/fibular + medial malleolus fracture/deltoid ligament injury); PLM-PER, trimalleolar fracture-pronation external rotation (posterior malleolus + lateral malleolus/fibular + medial malleolus fracture/deltoid ligament injury)

All 134 patients achieved fracture healing, and 39 patients (29%) showed positive ankle posttraumatic osteoarthritis. The average Olerud-Molander score was 89.9 (70,100), the average VAS score was 1.0 (0,5), and the ankle dorsiflexion was restricted by 4.0° (0°,9°) on the average (Table 1).

### Radiological prognosis (posttraumatic osteoarthritis) of posterior malleolar fractures with different step-off levels

The analysis of the posttraumatic osteoarthritis of posterior malleolar fractures with different step-off levels showed that compared with the positive rate of posttraumatic osteoarthritis at a postoperative step-off of < 1 mm, the positive rate of posttraumatic osteoarthritis was significantly higher when the continuous postoperative step-off of posterior malleolar fractures was 1–2 and > 2 mm (X^2^_1_ = 9.57, P1 = 0.002; X^2^_2_ = 10.75, P2 = 0.001). However, the difference in the positive rate of posttraumatic osteoarthritis between the postoperative step-offs of 1–2 and > 2 mm had no statistical significance (X^2^_3_ = 0.38, P3 = 0.536; Table 2).

### Functional prognosis (Olerud-Molander score, VAS score, and ankle dorsiflexion restriction) of posterior malleolar fractures with different step-off levels

**Fig. 4:**
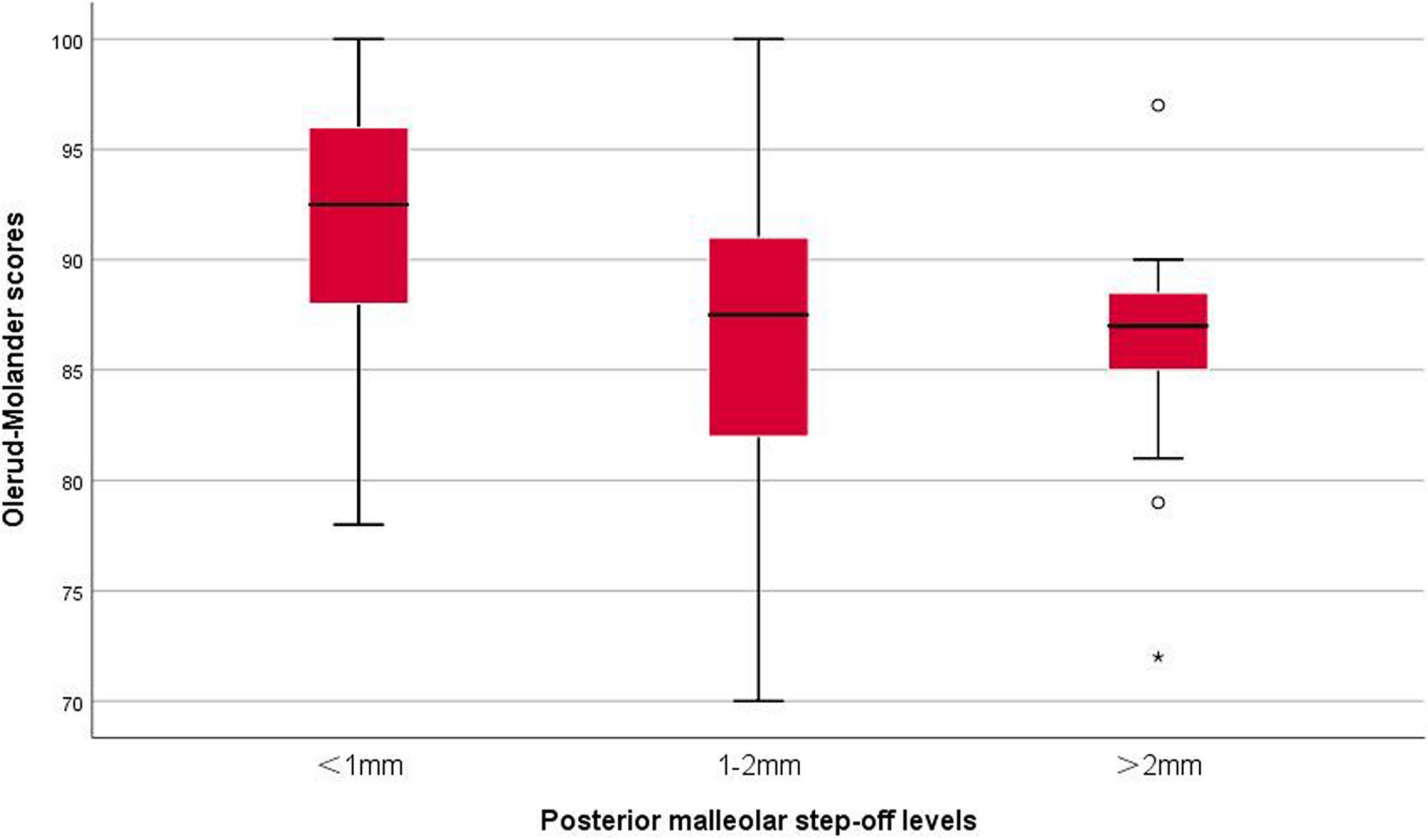
Olerud-Molander scores of posterior malleolar fractures with different step-off levels

The Olerud-Molander score, VAS score, and ankle dorsiflexion restriction of posterior malleolar fractures with different step-off levels showed similar trends. Compared with the functions of patients suffering from posterior malleolar fractures with a postoperative step-off of < 1 mm, those of patients suffering from posterior malleolar fractures with continuous postoperative step-offs of 1–2 and > 2 mm decreased significantly (Olerud-Molander: H1 = 11.24, P1 = 0.001 and H2 = 11.67, P2 = 0.001; VAS: H1 = 7.33, P1 = 0.007 and H2 = 11.35, P2 = 0.001; ankle dorsiflexion restriction: H1 = 3.98, P1 = 0.046 and H2 = 6.82, P2 = 0.009). However, no significant difference was observed in the patients’ functions with postoperative step-offs of 1–2 and > 2 mm (Olerud-Molander: H3 = 0.11, P3 = 0.746; VAS: H3 = 0.70, P3 = 0.404; ankle dorsiflexion restriction: H3 = 1.03, P3 = 0.310), as shown in Table 2; Fig. 4.

**Table 2:** Analysis of radiological results (osteoarthritis) and functional prognosis (Olerud-Molander score, VAS score, and ankle dorsiflexion restriction) of posterior malleolar fractures with different step-off levels

**Factor**	**Step-off level**			**Value**, P^*^
	**<1mm(N=88)**	**1-2mm(N=30)**	**>2mm(N=16)**	
**Osteoarthritis**				X^2^=15.29, P<0.001
Positive, N (%)	16 (18.2)	14 (46.7)	9 (56.3)	X^2^_1_=9.57,P1=0.002; X^2^_2_=10.75,P2=0.001; X^2^_3_=0.38,P3=0.536
**Olerud-Molander**				H=18.97,P<0.01
M (Q1, Q3)	93 (88,96)	88 (82,91)	87 (85,89)	H1=11.24,P1=0.001; H2=11.67,P2=0.001; H3=0.11,P3=0.746
**VAS**				H=14.77,P=0.001
M (Q1, Q3)	0 (0,1)	1 (0,3)	2 (0,3)	H1=7.33,P1=0.007; H2=11.35,P2=0.001; H3=0.70,P3=0.404
**Ankle dorsiflexion restriction**				H=9.13,P=0.010
M( Q1, Q3)	5 (0,5)	6 (2,7)	6 (3,8)	H1=3.98,P1=0.046 H2=6.82,P2=0.009 H3=1.03,P3=0.310

*H, X^2^，P: test value and *P* value in the comparison of three step-off levels; H1, X^2^_1_，P1: test value and *P* value of intergroup comparisons (step-offs of <1 and 1–2 mm); H2, X^2^_2_，P2: test value and *P* value of intergroup comparisons (step-offs of <1 and >2 mm); H3, X^2^_3_，P3: test value and *P* value of intergroup comparisons (step-offs of 1–2 and >2 mm)

### Subgroup analysis of posttraumatic osteoarthritis and Olerud-Molander scores of posterior malleolar fractures with different step-off levels

**Fig. 5:**
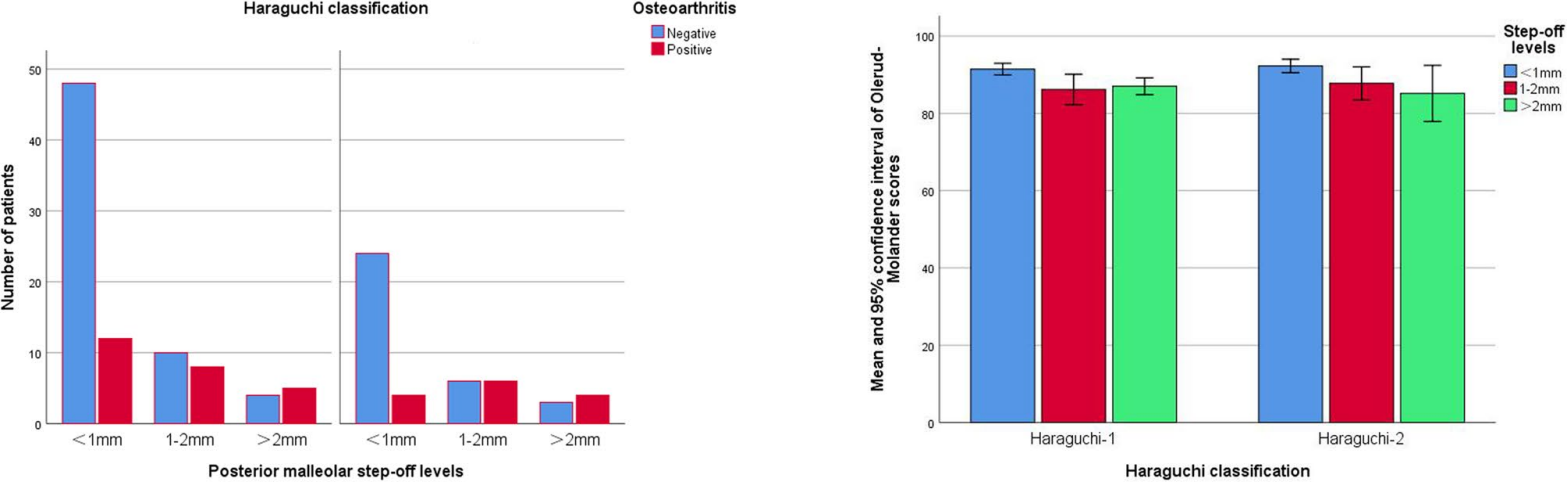
Subgroup analysis of posttraumatic osteoarthritis and Olerud-Molander scores of posterior malleolar fractures with different step-off levels

The Haraguchi-1 and Haraguchi-2 posterior malleolar fractures showed that the positive rate of posttraumatic osteoarthritis in patients with postoperative step-offs of 1–2 and > 2 mm was higher than that in patients with postoperative step-offs of < 1 mm (Haraguchi-1: X^2^_1_ = 4.34, P1 = 0.039 and X^2^_2_ = 5.33, P2 = 0.021; Haraguchi-2 type: X^2^_1_ = 5.71, P1 = 0.017 and X^2^_2_ = 5.83, P2 = 0.016). The Olerud-Molander score of patients with postoperative step-offs of 1–2 and > 2 mm was lower than that of patients with postoperative step-offs of < 1 mm (Haraguchi-1 type: H1 = 7.04, P1 = 0.008 and H2 = 6.45, P2 = 0.011; Haraguchi-2 type: H1 = 4.31, P1 = 0.038 and H2 = 4.73, P2 = 0.030), as shown in Table 3; Fig. 5.

**Table 3:** Subgroup analysis of osteoarthritis and Olerud-Molander score of posterior malleolar fractures with different step-off levels

Step-off level	Osteoarthritis		Olerud-Molander	
	Positive, *N*(%)	Value, P^*^	M(Q1, Q3)	Value, P^*^
**Haraguchi-1**		X^2^ = 7.57,*P* = 0.023		H = 11.33,*P* = 0.003
<1 mm(*N* = 60)	12(20.0)	X^2^_1_ = 4.34,P1 = 0.039	92(88,96)	H1 = 7.04,P1 = 0.008
1–2 mm(*N* = 18)	8(44.4)	X^2^_2_ = 5.33,P2 = 0.021	87(82,90)	H2 = 6.45,P2 = 0.011
>2 mm(*N* = 9)	5(55.6)	X^2^_3_ = 0.30,P3 = 0.586	87(86,90)	H3 = 0.13,P3 = 0.717
**Haraguchi-2**		X^2^ = 8.07,*P* = 0.018		H = 7.37,*P* = 0.025
<1 mm(*N* = 28)	4(14.3)	X^2^_1_ = 5.71,P1 = 0.017	93(88,96)	H1 = 4.31,P1 = 0.038
1–2 mm(*N* = 12)	6(50.0)	X^2^_2_ = 5.83,P2 = 0.016	90(83,94)	H2 = 4.73,P2 = 0.030
>2 mm(*N* = 7)	4(57.1)	X^2^_3_ = 0.09,P3 = 0.764	87(79,88)	H3 = 0.41,P3 = 0.525

* H, X^2^, P: test value and *P* value in the comparison of three step-off levels; H1, X^2^_1_, P1: test value and *P* value of intergroup comparisons (step-offs of < 1 and 1–2 mm); H2, X^2^_2_, P2: test value and *P* value of intergroup comparisons (step-offs of < 1 and > 2 mm); H3, X^2^_3_, P3: test value and *P* value of intergroup comparisons (step-offs of 1–2 and > 2 mm)

### Risk factors for ankle posttraumatic osteoarthritis

In the univariate analysis, age, area ratio of posterior malleolar fragments, and posterior malleolar step-off were potential risk factors of posttraumatic osteoarthritis (*P* < 0.20) and included in the multivariate logistic regression model. The results showed that only posterior malleolar step-off was a statistically significant independent risk factor for posttraumatic osteoarthritis (Wald = 14.23, *P* < 0.01; Table 4).

**Table 4:** Risk factor analysis of radiological results (osteoarthritis) and functional prognosis (Olerud-Molander score)

**Factor**	**Univariable analysis**		**Multivariable analysis**		
	**Value**	***P***	**Value**	***P***	**95% Confidence Interval**
**(A) O** **steoarthritis**					
Age	2.73	0.099	3.10	0.078	2.139(0.918,4.985)
Haraguchi classification	0.02	0.898	-	-	
Area ratio of the posterior malleolar fragment	1.86	0.173	1.89	0.170	2.115(0.726,6.162)
Posterior malleolar step-off	15.29	＜0.01	14.23	＜0.01	3.003(1.696,5.316)
**(B) ** **Olerud-Molander** ** score**					
Age	1.08	0.300	-	-	
Haraguchi classification	0.56	0.457	-	-	
Area ratio of the posterior malleolar fragment	2.67	0.105	−1.84	0.069	−2.184(−4.539,0.171)
Posterior malleolar step-off	7.51	＜0.01	−3.55	＜0.01	−2.682(−4.178,−1.187)
Osteoarthritis	5.35	0.022	−2.62	0.010	−3.061(−5.374,−0.748)

### Risk factors for clinical functional prognosis (Olerud-Molander) of the ankle

In the univariate analysis, the area ratio of posterior malleolar fragments, posterior malleolus step-off, and post-traumatic osteoarthritis were the potential risk factors leading to low Olerud-Molander scores (*P* < 0.20). In the multivariate analysis, only posterior ankle step-off and post-traumatic osteoarthritis were independent risk factors for poor functional prognosis (*P* < 0.05; Table 4.).

## Discussion

The treatment and prognosis of trimalleolar fractures, especially posterior malleolar fractures, is still the focus of debate among trauma orthopedic surgeons [2, 19, 20]. The risk factors that affect the prognosis of posterior malleolar fractures reported in previous related studies are different, and the conclusions are inconsistent or even contradictory [11, 21, 22].

In this study, the effects of persistent posterior malleolar fractures with different step-off levels on the radiological results and functional prognosis were analyzed for the first time. Compared with previous similar studies, this study further compared and analyzed the difference in the prognosis of different step-off levels. Another advantage of this study is that the methods of Lee SH et al. [15] and Marques RH et al. [16] (CT scanning) were used to measure the percentage of posterior malleolar fracture on the articular surface and the step-off of posterior malleolar fracture (with the maximum value of the CT sagittal plane as the measurement standard). The measured values were accurate, which is particularly important for the minor differences in fracture reduction quality. In previous related studies, X-ray films were mostly used to measure posterior malleolar fracture [9, 13]. Ferries J.S. et al. [23] and Verhage S.M. et al. [24] believed that the consistency of X-ray films in measuring posterior malleolar fracture among observers is poor. The related measurement errors may lead to a decrease in the reliability of the corresponding clinical and prognostic significance.

### Posterior malleolar fracture and ankle posttraumatic osteoarthritis

The general belief in orthopedics is that the remaining step-offs during intra-articular fracture healing increase the incidence of posttraumatic osteoarthritis, and restoring the congruity of the articular surface is the key to dealing with this kind of injury [25–27]. Verhage S.M. et al. [13] and Drijfhout van Hooff C. C. et al. [9] reported that when the postoperative step-off of posterior malleolar fracture reaches or exceeds 1 mm, the incidence of radiographic posttraumatic osteoarthritis is high regardless of whether the posterior malleolar fragment is fixed or not. After analyzing the risk factors for postoperative posttraumatic osteoarthritis of trimalleolar fractures, Xie W. Y. et al. [12] concluded that poor reduction of posterior malleolar fractures is an independent risk factor for postoperative posttraumatic osteoarthritis (*P* < 0.05), the incidence of which can be reduced by improving the reduction quality of posterior malleolar fractures. The conclusion of our study coincides with that of previous studies, namely, the positive rate of posttraumatic osteoarthritis is significantly high when the postoperative step-off of posterior malleolar fractures is > 1 mm. In the case of poor reduction of posterior malleolar fractures (postoperative step-off of > 1 mm), the proportion of postoperative posttraumatic osteoarthritis among patients increased. However, in the case of nonanatomical reduction of posterior malleolar fractures (postoperative step-off of > 1 mm), the increase in the posterior malleolar step-off did not further increase the incidence of posttraumatic osteoarthritis.

A regression analysis was further conducted and showed that only posterior malleolar step-off was an independent risk factor for posttraumatic osteoarthritis (*P* < 0.01; Table 4.). However, the area ratio of the posterior malleolar fragment was not a risk factor (*P* = 0.170), which is consistent with the conclusions of Verhage S. M. et al. [12] and Neumann A. P. et al. [10] but contradicts those of Drijfhout van Hooff C. C. et al. [8] and Abarquero-Diezhandino et al. [9]. The added Haraguchi classification analysis in this study revealed that it was a nonindependent risk factor, suggesting that no statistical difference existed in the incidence of post-traumatic osteoarthritis between Haraguchi-1 and Haraguchi-2 types.

### Posterior malleolar fracture and clinical functional prognosis of the ankle

The treatment of posterior malleolar fractures aims to achieve anatomical reduction of fragments, restoration of the congruency of articular surfaces, and stability of the tibiotalar joint and tibiofibular syndesmosis [28–30]. Most studies believed that poor fracture reduction can lead to ankle fracture malunion, pain, and limitation in movement [7, 9]. Verhage S. M. et al. [13] reported that a postoperative step-off greater than 1 mm for posterior malleolar fractures is an independent risk factor for posttraumatic osteoarthritis, which is an independent risk factor for poor functional prognosis. Xie W.Y. et al. [12] found that poor reduction of posterior malleolar fractures and a posttraumatic osteoarthritis score greater than 1 are independent risk factors for postoperative functional prognosis of trimalleolar fractures (*P* < 0.05). Improving the reduction quality of posterior malleolar fragments can reduce the incidence of long-term posttraumatic osteoarthritis after trimalleolar fracture operation and improve the function of the affected limb. Inconsistent conclusions about the prognostic significance of the posterior malleolar fracture step-off have also been obtained. Drijfhout van Hooff C. C. et al. [9] stated that when the postoperative step-off of posterior malleolar fractures reaches or exceeds 1 mm, although the incidence of radiographic posttraumatic osteoarthritis increases, no statistical difference is observed in the clinical score.

The conclusion of our study is consistent with those of most studies, such as the study of Verhage S.M. et al. [13], which concluded that poor reduction of posterior malleolar fractures (postoperative step-off > 1 mm) affects the functional prognosis of the affected limb (decreased Olerud-Molander score). When poor reduction of posterior malleolar step-offs was further divided in the current study, no statistically significant differences were observed in the functions of patients between postoperative step-offs of 1–2 and > 2 mm. Therefore, in clinical work, the focus should still be on the anatomical reduction of posterior malleolar fractures to improve the long-term functional efficacy of patients. The regression analysis in this study revealed that Haraguchi classification and area ratio of posterior malleolar fractures were not risk factors for functional prognosis (*P* > 0.05), a result that is consistent with the conclusions of Verhage S. M. et al. [13], Drijfhout van Hooff C. C. et al. [9], and Neumann A. P. et al. [11]. This study showed that controlling the posterior malleolar fracture during operation to achieve anatomical reduction is still the key to improving the long-term curative effect on patients. Regardless of the size and classification of posterior malleolar fragments, as long as the quality of fracture reduction is increased, the functional prognosis of patients can be improved, and the medium and long-term curative effects can be predicted.

### Limitations of this study

The retrospective research method was adopted for data collection and analysis, which is the main limitation of this study. Moreover, the follow-up period of some patients was short, which may lead to an underestimation of the incidence of post-traumatic arthritis. Continuous follow-up visits are expected in the future to establish a long-term follow-up database of patients.

## Conclusion

Posterior malleolar step-off is an independent risk factor for ankle posttraumatic osteoarthritis, and poor reduction of posterior malleolar fractures and posttraumatic osteoarthritis are associated with poor functional prognosis of the ankle. The future clinical emphasis should still be on the anatomical reduction of posterior malleolar fractures to improve the long-term functional efficacy of patients.

## Data Availability

The data sets supporting the conclusion of this article are included in the manuscript. Upon request, raw data can be provided by the corresponding author.
